# Efficacy of Contractility Modulation Therapy in Patients with Transthyretin Amyloid Cardiomyopathy, Mildly Reduced to Reduced EF and NYHA III and IV: A Multicentric, Prospective Pilot Study for AMY-CCM Registry

**DOI:** 10.3390/jcdd12100380

**Published:** 2025-09-25

**Authors:** Procolo Marchese, Francesca Gennaro, Giovanni Mazzotta, Pierfrancesco Grossi, Luigi Cocchiara, Stefano Guarracini, Lorenzo Mazzocchetti, Matteo Ziacchi, Mauro Biffi, Roberta Magnano, Massimo Di Marco, Matteo Ruzzolini, Antonio Bisignani, Matteo Bianco, Paolo Garrone, Walter Grosso Marra, Margherita Cannillo, Carlo Lavalle, Daniele Masarone, Cristina Chimenti

**Affiliations:** 1 Cardiology Unit, Mazzoni Civil Hospital, AST Ascoli Piceno, 63100 Ascoli Piceno, Italy; francescagennaro@hotmail.com (F.G.); mazzottagio@gmail.com (G.M.); p.f.grossi@gmail.com (P.G.); 2 Department of Advanced Biomedical Sciences, University of Naples Federico II, 80131 Naples, Italy; luigicocchiara@libero.it; 3 Department of Cardiology, “Pierangeli” Hospital, 65129 Pescara, Italy; stefanoguarracini@yahoo.it (S.G.); lorenzomazzocchetti90@gmail.com (L.M.); 4 Cardiology Unit, Cardiac Thoracic and Vascular Department, IRCCS Azienda Ospedaliero-Universitaria di Bologna, Via Giuseppe Massarenti 9, 40138 Bologna, Italy; matteo.ziacchi@gmail.com (M.Z.); mauro.biffi@aosp.bo.it (M.B.); 5 Cardiology Unit, Ospedale Civile Santo Spirito, 65100 Pescara, Italy; robertamgn@live.it (R.M.); massimo.dimarco@asl.pe.it (M.D.M.); 6 Department of Cardiovascular and Thoracic Sciences, Ospedale Isola Tiberina-Gemelli Isola, 00186 Rome, Italy; matteo.ruzzolini@gmail.com (M.R.); abisignani@hotmail.it (A.B.); 7 SCDO Cardiology, Azienda Ospedaliera Universitaria San Luigi Gonzaga, 10043 Orbassano, Italy; matteo.bianco87@gmail.com (M.B.); p.garrone@hotmail.com (P.G.); 8 Division of Cardiology, Ivrea Hospital, ASLTO4, 10015 Ivrea, Italy; grossomarra@yahoo.it (W.G.M.); margheritacannillo@gmail.com (M.C.); 9 Department of Internal Clinical, Anesthesiological and Cardiovascular Sciences, “Sapienza” University of Rome, 00185 Rome, Italy; carlolavalle@yahoo.it (C.L.); cristina.chimenti@uniroma1.it (C.C.); 10 Heart Failure Unit, Department of Cardiology, AORN dei Colli/Monaldi Hospital, 80131 Naples, Italy; danielemasarone@gmail.com

**Keywords:** amyloidosis, cardiac contractility modulation, heart failure

## Abstract

Background: Transthyretin cardiac amyloidosis (ATTR-CM) is an infiltrative cardiomyopathy that frequently progresses to symptomatic heart failure (HF), often with mildly reduced or reduced ejection fraction (EF). Standard therapies are limited in NYHA III–IV, and Tafamidis is approved only for the early stages. Cardiac contractility modulation (CCM) therapy has shown promise in HF with reduced EF, but its role in ATTR-CM remains unexplored. Methods: This multicentric, prospective pilot study evaluated the safety and efficacy of CCM therapy in ten patients (*n* = 10) with ATTR-CM, EF between 25 and 45%, and NYHA class III–IV symptoms refractory to optimal medical therapy. All patients underwent implantation of the Optimizer CCM system and were followed for at least 12 months. The primary endpoint was the incidence of worsening heart failure (WHF); secondary endpoints included changes in EF, NYHA class, 6-minute walk test (6MWT), and quality of life metrics. Results: In this cohort (*n* = 10), CCM therapy significantly reduced WHF episodes (from 0.18 ± 0.09 to 0.025 ± 0.08 hospitalizations/patient-year, *p* < 0.001) and improved NYHA class and 6MWT (*p* < 0.001). EF increased by an average of 4.8 ± 6.1%, and 6MWT improved by 31.3 ± 53.3%. Importantly, all patients became eligible for Tafamidis after CCM therapy due to improved functional status. Conclusion: This pilot study suggests that CCM therapy is a feasible and potentially effective option for ATTR-CM patients with advanced HF who are not candidates for existing disease-modifying treatments. These findings support the rationale for larger studies, including the ongoing AMY-CCM registry (NCT05167799), to validate CCM’s therapeutic role in this population.

## 1. Introduction

Transthyretin cardiac amyloidosis (ATTR-CM) is a progressive infiltrative cardiomyopathy caused by the extracellular deposition of misfolded transthyretin protein in the myocardium. ATTR-CM is increasingly recognized as an underdiagnosed cause of heart failure in older adults, typically manifesting with a restrictive/hypertrophic cardiomyopathy phenotype. The progression of this disease can result in the development of HF, characterized by a preserved ejection fraction, a mildly reduced ejection fraction, or a reduced ejection fraction [1].Notwithstanding, the condition has been linked to severe aortic stenosis and atrial fibrillation.

In advanced ATTR-CM, conventional heart failure therapies are frequently ineffective or poorly tolerated, primarily due to adverse events such as bradycardia and hypotension. Consequently, contemporary ESC guidelines do not endorse the systematic use of β-blockers, ACE inhibitors, or ARBs in this population, and instead recommend reliance on diuretics for symptomatic relief. The role of β-blockers remains controversial, with some data suggesting potential harm in amyloid cardiomyopathy, while the use of ACEIs/ARBs is discouraged due to hypotension, and diuretics are recommended for symptom control [2].

The prevailing treatment recommendations encompass the use of genetic silencers, stabilizers, and removers. The efficacy of these therapeutic interventions has been demonstrated in the reduction in mutated TTR production (liver transplantation) and the stabilization of circulating TTR molecules (genetic silencers), thereby preventing their dissociation or cleavage into amyloidogenic fragments. Tafamidis, a stabilizer, is regarded as the primary treatment option for ATTR cardiac patients with a reasonable expected survival prognosis [3]. The prescription of other medications, including Patisiran and Inotersen, is exclusively indicated for patients diagnosed with polyneuropathy resulting from amyloid accumulation [2]. However, the administration of Tafamidis is currently restricted to patients with NYHA class I and II. This limitation results in a significant gap in the management of ATTR-CM patients with NYHA III/IV or who do not respond to Tafamidis treatment and do not have polyneuropathy. In such cases, heart transplantation remains the sole viable management strategy.

This therapeutic uncertainty underscores a major gap in the management of patients with advanced ATTR-CM, particularly those with NYHA III–IV symptoms and reduced ejection fraction, who are also excluded from tafamidis treatment.

Against this background, cardiac contractility modulation (CCM) therapy may represent an innovative option. CCM delivers non-excitatory biphasic electrical impulses during the absolute refractory period of the myocardial action potential. It has been shown to improve symptoms and outcomes in patients with HFrEF despite optimal medical therapy, specifically in the EF range of 25–45%.

The mechanism of CCM involves both acute enhancement of contractility via calcium handling proteins and longer-term reverse remodeling through gene expression changes and these mechanisms may be particularly relevant in ATTR-CM, where conventional therapies are often poorly tolerated due to bradyarrhythmia or hypotension [4]. Given the overlap between the pathophysiological derangements targeted by CCM and those observed in ATTR-CM, this study was designed to explore the feasibility, safety, and potential clinical benefits of CCM in a cohort of ATTR-CM patients with NYHA class III–IV symptoms and moderately to severely reduced EF. Importantly, these patients represent a therapeutic gap in current clinical practice, as they are typically excluded from both pharmacological and conventional device-based therapies. This pilot investigation serves as an initial step toward evaluating the role of CCM as an adjunctive or bridging therapy in this high-risk population.

## 2. Materials and Methods

### 2.1. Study Design and Population

This was a prospective, multicenter pilot study designed to evaluate the clinical feasibility, safety, and potential efficacy of CCM therapy in patients with ATTR-CM, presenting with symptomatic heart failure and reduced or mildly reduced ejection fraction. The study was conducted across eight Italian cardiology centers with established expertise in heart failure and device therapies, ensuring standardized procedural protocols and data collection methods. The diagnosis of ATTR-CA was made in accordance with the recommendations established by the ESC [2]. No patients required endomyocardial biopsy. Following the provision of informed consent, all patients underwent Optimizer Smart System (Impulse Dynamics Inc., Marlton, NJ, USA) device implantation as part of routine clinical care, utilizing the previously described technique [5], the implant was performed by expert physicians. CCM therapy is a proper HF therapy and delivers high amplitude non-excitatory biphasic electrical signals during the myocardial refractory period. The CCM implant is similar to pacemaker implantation, except that two RV leads are placed instead of one. The procedure is performed using cephalic or subclavian vein access, with the former being the more common approach. It should be noted that the right side is often selected as the access site, given the prevalence of existing implantable cardioverter-defibrillators (ICDs) on the left side. Two active fixation leads are secured to the right ventricular septum at least 2–3 cm apart from each other and at least 3 cm from the defibrillation RV lead. Leads were required to sense ventricular activity and deliver CCM signal. Electrical testing of the leads involves the standard testing procedures for pacemaker leads, with an emphasis on the sensing function. Active CCM treatment is typically scheduled to be administered daily for at least seven hours, distributed evenly throughout the day, with each interval measuring one hour. The objective is to ensure a minimum of 90% completion of the CCM therapy [5,6,7,8,9,10]. Following implantation, CCM therapy was initiated with daily sessions of at least seven hours, scheduled in one-hour intervals to optimize myocardial conditioning while allowing for rest periods. Device interrogation was performed before discharge and at each follow-up visit to verify lead integrity, sensing thresholds, and therapy adherence (targeting >90% daily delivery). While implantation techniques followed a uniform protocol across centers, CCM output parameters (pulse amplitude, width, and duty cycle) were initially programmed based on manufacturer guidelines and could be individualized according to patient response.

Patients were followed at 1, 3, 6, and 12 months, with clinical evaluations, echocardiographic assessment, device checks, and completion of functional tests including the 6-minute walk test (6MWT) and the Kansas City Cardiomyopathy Questionnaire (KCCQ). Any hospitalizations or episodes of WHF were documented in a central registry. Demographic data, medical history, adverse events, additional clinical and echocardiographic data were recorded both at baseline and at follow-up visits.

Inclusion criteria, from AMY-CCM Registry (NCT05167799), were: (1) HF with mid-range to reduced EF and diagnosis ATTR-CM; (2) NYHA class III–IV (despite optimal medical therapy); (3) At least one episode of WHF in the previous 12 months (defined as hospitalization and/or acute intravenous interventions).

Exclusion criteria were: (1) AL amyloid cardiomyopathy (as it represents a different clinical setting); (2) Patients with a potentially correctible cause of HF; (3) Patients requiring CABG or PCI; (4) Patients who have undergone a CABG within 90 days or PCI within 30 days; (5) Patients who have experienced a myocardial infarction within the past 90 days; (6) Mechanical tricuspid valve; (7) Prior heart transplant; (8) Chronic hemodialysis; (9) Patients with familial TTR amyloidotic cardiomyopathy with significant polyneuropathy potentially eligible for Patirisan or Inotersen.

The primary endpoint of the study was the incidence of WHF.

The secondary endpoints were ejection fraction (EF), 6MWT, NYHA class and KCCQs changes after CCM implant.

This study was approved by the institutional review boards of all participating centers and conducted in accordance with the Declaration of Helsinki. All patients provided written informed consent for both treatment and data usage.

### 2.2. Definitions

WHF was defined as deterioration of signs and symptoms of HF that requires IV diuretic therapy. EF was calculated using automatic quantification of end-diastolic volume (EDV) and end-systolic volume (ESV). 6MWT was defined as the distance covered by walking for over 6 min. NYHA class was calculated during the patient interview and according to the guidelines [11]. KCCQs was calculated according to Spertus et al. [12].

### 2.3. Statistical Analysis

All statistical analyses were performed using SPSS software version 22 (IBM Corp., Armonk, NY, USA). Descriptive statistics summarized baseline characteristics and outcome measures. Continuous variables were reported as mean values with interquartile ranges (IQR), while categorical variables were expressed as frequencies and percentages. Comparisons of categorical variables were made using the Chi-square test. For continuous variables, paired t-tests were applied to compare pre- and post-treatment values. All tests were two-sided, with a significance level set at *p* < 0.05.

## 3. Results

### 3.1. Study Population

A total of ten patients meeting the predefined inclusion criteria were enrolled and successfully underwent implantation of the Optimizer CCM device. The demographic and baseline clinical characteristics of the study population are detailed in Table 1. The mean age was 76.7 ± 4.8 years, with the majority being male (90%, *n* = 9). All patients had a confirmed diagnosis of ATTR-CM, with the wild-type form accounting for 90% of cases (*n* = 9), and one patient carrying a hereditary mutation. The cohort displayed a high prevalence of cardiovascular comorbidities: hypertension was present in 90% of patients, diabetes in 50%, and atrial fibrillation in 50%. Chronic kidney disease (eGFR < 60 mL/min/1.73 m^2^) affected 70% of patients, and 40% had pericardial effusion identified on baseline echocardiography (Table 1). All subjects met the inclusion criteria, which stipulated that they receive optimal medical therapy. Notably, baseline NYHA class was 3.2 ± 0.4, with a mean EF of 36.5 ± 7.3%. Despite receiving optimized medical therapy, including loop diuretics (100%) and beta-blockers (60%), all patients experienced at least one episode of WHF in the 12 months preceding CCM implantation (mean: 2.0 ± 1.2 events/year). Only three patients (30%) were on Tafamidis at the time of enrolment, but without demonstrable clinical benefit (see Table 2 and Table 3). Baseline functional and laboratory parameters are summarized in Table 4. Patients presented with advanced disease, as indicated by the median NYHA class, markedly impaired exercise capacity (6MWT 265 ± 100 m), reduced quality-of-life scores (KCCQ 42 ± 31), and markedly elevated NT-proBNP levels (4301 ± 3086 pg/mL). Echocardiography confirmed a restrictive phenotype, with thickened ventricular walls (IVS 21.3 ± 3.5 mm, posterior wall 18 ± 6.7 mm), reduced longitudinal strain (7.5 ± 1.5%), and evidence of RV involvement (TAPSE 12.2 ± 2.3 mm). Notably, pericardial effusion was present in 40% of patients. Procedural safety was prospectively monitored; no peri-procedural complications were observed, including lead dislodgement, infections, hematoma, or pneumothorax.

### 3.2. Primary Outcome

**Figure 1:**
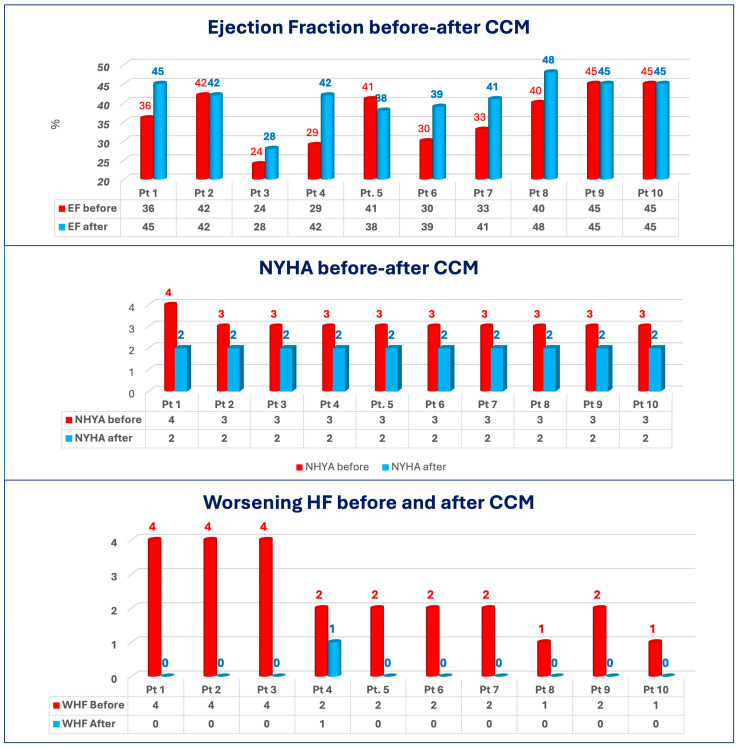
WHF, NYHA class and EF before and after CCM implantation.

Following a minimum 12-month follow-up period (range: 12–42 months), there was an 86% reduction in WHF episodes compared to the baseline (0.025 ± 0.08 vs. 0.18 ± 0.09, *p* = 0.001). As illustrated in Figure 1 and Table 5, among the 10 patients (all experiencing multiple episodes of WHF yearly), only one patient exhibited a single episode of WHF after CCM implantation.

### 3.3. Secondary Outcome

In the entire cohort, there has been an improvement in Ejection Fraction as well as NYHA Class (Table 5 and Figure 1). In addition to reducing HF-related hospitalizations, CCM therapy was associated with marked improvements in multiple secondary clinical and functional endpoints (Table 5 and Table 6 and Figure 1):

NYHA Functional Class: All patients experienced a one-class improvement, transitioning from NYHA class III to class II by the end of follow-up. This improvement was uniform, with no inter-patient variability, highlighting the consistent improvement in symptoms with CCM (mean NYHA class change: −1.2 ± 0.4; *p* < 0.001).

Left Ventricular Ejection Fraction (EF): Mean EF increased from 36.5 ± 7.3% at baseline to 41.3 ± 7.6% at follow-up, reflecting a mean absolute improvement of 4.8 ± 6.1%. While modest, this increase is clinically relevant, particularly in patients with restrictive physiology where large changes are uncommon.

6-Minute Walk Test (6MWT): Baseline exercise tolerance was markedly impaired (mean 6MWT: 264.6 ± 100.1 m). Following CCM therapy, the mean distance increased by 31.3 ± 53.3%, demonstrating improved functional capacity. Notably, improvements were observed across all age groups, including the elderly with limited baseline mobility.

Kansas City Cardiomyopathy Questionnaire (KCCQ): Quality-of-life assessment via the KCCQ showed a substantial increase from a baseline mean of 42.0 ± 31.0 to 93.0 ± 38.5 (mean change: 121.0 ± 252.8%), reflecting meaningful enhancements in perceived physical limitations, symptom burden, and social function.

Diastolic Function and RV Performance: Echocardiographic indices also showed trends toward improvement. Mean TAPSE increased by 5.3 ± 10.3%, while the E/E′ ratio declined by −11.1 ± 22.7%, suggesting improved diastolic function.

### 3.4. Tafamidis Eligibility After CCM

An additional clinically significant observation was that, post-CCM, all patients met functional criteria for Tafamidis initiation based on improved NYHA classification (now class II in all). Among the seven patients who were not previously on Tafamidis, all began therapy within 4 to 8 weeks following CCM implantation, once NYHA class improved to II and eligibility criteria were met. This shift implies that CCM may not only provide direct benefits but could also facilitate access to disease-modifying therapy previously contraindicated due to symptomatic severity.

## 4. Discussion

### 4.1. Clinical Implications of CCM in ATTR-CM

In this pilot study, we demonstrated a beneficial effect of CCM in patients with HF related to ATTR-CM. Specifically, in this highly selected cohort of patients with reduced to mildly reduced left ventricular ejection fraction and NYHA class III–IV symptoms refractory to optimal medical therapy, CCM was associated with a marked reduction in heart failure hospitalizations, functional improvement, and enhanced quality of life.

Despite the advancements in medical and device therapy, HF remains a leading cause of mortality, partly because the majority of patients receiving OMT exhibit limited potential for up-titration. It is important to note that ICD does not provide therapeutic benefit for heart failure itself. However, only about one-third of HF patients meet CRT criteria, and one-third of CRT recipients do not respond. Consequently, these patients continue to experience symptoms despite undergoing OMT [10].

To our knowledge, this is the first prospective multicenter study specifically targeting CCM therapy in the ATTR-CM population. While CCM has been extensively validated in non-amyloid HFrEF populations, its application in infiltrative cardiomyopathies has remained largely unexplored. This study fills an important clinical gap and supports the hypothesis that the mechanistic benefits of CCM (enhanced inotropy, improved lusitropy, and reverse remodeling, achieved through the modulation of myocardial contraction and relaxation, which results in improvements in reported symptoms and a reduction in HF hospitalizations [6,9,13]) may translate into meaningful clinical benefits even in restrictive myocardial conditions such as ATTR-CM.

It is imperative to acknowledge that while all patients became eligible for Tafamidis therapy after CCM implantation, the observed clinical improvements, specifically in NYHA class, ejection fraction, functional capacity, and reduction in heart failure hospitalizations, cannot be attributed to Tafamidis itself. Tafamidis necessitates a protracted treatment period to yield clinical efficacy. The pivotal ATTR-ACT trial evidenced substantial reductions in all-cause mortality and cardiovascular-related hospitalizations only after a minimum of 18 months of continuous therapy [14]. Conversely, subsequent evaluations revealed considerable enhancements as early as 12 months following CCM implantation, well before Tafamidis’s anticipated therapeutic action. Consequently, the observed improvements in this cohort are most plausibly associated with the physiological benefits of CCM therapy rather than with Tafamidis administration. Indeed, by improving symptoms and functional capacity, CCM may serve as a therapeutic bridge or adjunct to pharmacologic therapy, potentially reshaping current treatment algorithms for this challenging disease.

The improvements in left ventricular ejection fraction, though modest, align with data from non-amyloid HFrEF trials, suggesting a consistent physiological response. Likewise, the significant gain in 6-minute walk distance and the dramatic improvement in KCCQ scores reinforce the positive impact of CCM, addressing symptoms, physical function, and overall quality of life.

Evidence from larger non-amyloid HFrEF cohorts suggests that patients with baseline EF ≥ 35% derive more pronounced benefits compared to those with EF < 35% [7,10]. Our population spanned this range, and while all patients improved, future larger studies should stratify by EF to assess differential response in ATTR-CM.

### 4.2. Mechanistic Rationale for CCM in Amyloidosis

**Figure 2:**
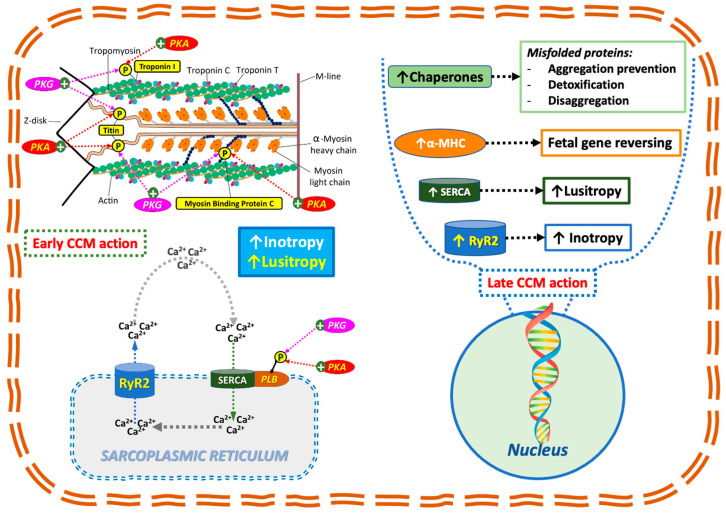
Effects of CCM. Schematic representation of early and late onset effect on CCM function (see explanation in the text). Left side of the picture refers to early onset effect which are related to enzyme modulation by CCM induced EMF. Increase in phosphorylation state of troponin and Myosin Binding Protein C leads to positive inotropy. Increase in phosphorylation state of PLB and Titin leads to positive lusitropy. PKA: phosphokinase A; PKB: phosphokinase B. Right side of the picture refers to late onset effects which are related to DNA transcription modulation by CCM induces EMF. There is a substantial fetal gene reverse remodeling by increasing the down regulated RyR2, SERCA and α-MHC. Moreover, the increase in Chaperones transcription (such as HSP70) has several positive effects such as aggregation prevention, detoxification and disaggregation of misfolded proteins (Reprinted with permission from Ref. [4], Copyright 2003 J. Clin. Med.).

**Figure 3:**
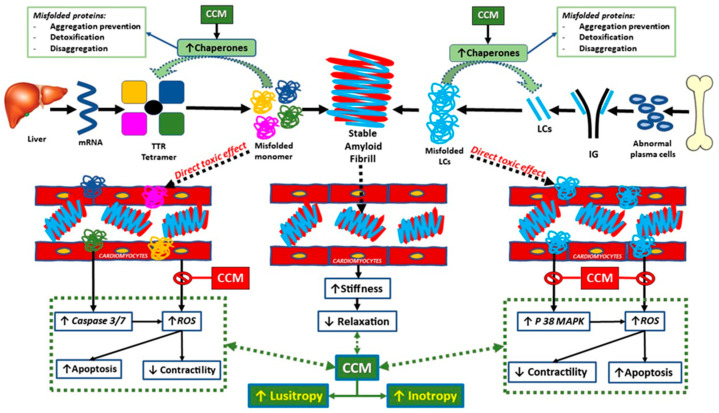
CCM effect in ATTR-CM. Cardiac Amyloid pathophysiology and CCM therapeutic effect. See explanation in the text. TTR: transtiretin. CCM: cardiac contractility modulation; LC: light Chains; IG: immune globulin; ROS: reactive oxygen species; P 38 MAPK: p38 mitogen activated protein-kinase (Reprinted with permission from Ref. [4], Copyright 2003 J. Clin. Med.).

In our first report, we summarized the CCM “pharmacodynamic” [4] which appears to be consistent with the pathophysiology associated with ATTR-CM. CCM acts in an early onset fashion by increasing phosphorylation of troponin and Myosin Binding Protein C [15], which results in a positive inotropy; it also increases the phosphorylation state of PLB [16] and Titin, which induces positive lusitropy [8] (Figure 2). Furthermore, CCM has also been demonstrated to exert late-onset effects through EM field action on specific DNA sequences [17], which have been shown to reverse maladaptive fetal gene remodeling [18]. This process has been demonstrated to regulate the expression of key sarcoplasmic reticulum genes by increasing the expression of RyR2, SERCA, and α-MHC, which had previously been found to be downregulated. Furthermore, the augmentation in chaperone transcription (e.g., HSP70) engenders numerous advantageous outcomes, including the prevention of aggregation, detoxification, and the disaggregation of misfolded proteins^4^ (Figure 3). A growing body of research has demonstrated that CCM positively influences several processes associated with amyloid cardiomyopathy. CCM-driven normalization of elevated diastolic Ca^2+^ levels in the failing heart might be related to ROS reductions and activation of CaMKII [5]. CCM has been demonstrated to decrease the expression of p38 mitogen-activated protein kinase (p38MAPK). This particular protein is involved in the direct toxic amyloidogenic-mediated oxidative stress, dysfunction, and cell death of cardiomyocytes [19] (see Figure 2).

It has been demonstrated that CCM substantially influences the transcription of chaperones, including HSP70. The function of these chaperones is to facilitate the equilibrium between protein synthesis and degradation. They aid in the process of refolding misfolded proteins and protect cells against death in conditions that are stressful or pathological, such as amyloid [20].

It is important to note that the present study did not include systematic evaluation of myocardial fibrosis by cardiovascular magnetic resonance (CMR) or histopathology. The extent of fibrosis and viable myocardium are likely major determinants of the response to CCM in ATTR-CM. Without these data, our findings cannot establish mechanistic explanations for the observed benefits. Instead, this pilot study should be interpreted as hypothesis-generating, showing feasibility and safety while raising the need for fibrosis-stratified studies.

The validity of these data is contingent upon validation from the ongoing AMY-CCM registry (ClinicalTrials.gov Identifier: NCT05167799). The registry’s findings have the potential to provide novel evidence regarding the impact of CCM therapy in cardiac amyloidosis, specifically as a synergistic therapeutic option.

Nevertheless, the precise pathways through which CCM improves outcomes in amyloidosis remain incompletely understood, and mechanistic studies integrating imaging, biomarkers, and molecular endpoints are warranted.

## 5. Conclusions

In this multicenter prospective pilot study, CCM therapy appeared feasible and safe, with consistent clinical improvements in patients with advanced ATTR-CM and reduced/mid-range EF. By reducing hospitalization rates, enhancing functional status, and enabling eligibility for Tafamidis, CCM might represent a valuable therapeutic option for a population with few alternatives. However, given the small sample size and absence of fibrosis imaging, these results should be considered hypothesis-generating. Larger studies incorporating CMR assessment are warranted to confirm the benefits and define the optimal role of CCM in amyloid cardiomyopathy.

## 6. Limitations

Despite these encouraging results, several limitations must be acknowledged. The sample size was small, reflecting the pilot nature of the study, and the absence of a control group limits causal inference. However, the magnitude and consistency of improvement across multiple endpoints, including objective and patient-reported outcomes, support a true treatment effect. In addition, the follow-up duration may not have been sufficient to observe the full effects of Tafamidis in patients who initiated it post-CCM, though early functional gains are unlikely to be attributable to Tafamidis alone, given its delayed onset of action. A key limitation of this study is the absence of systematic CMR data or endomyocardial biopsy, which precluded assessment of myocardial fibrosis and viable cardiomyocyte burden. As a result, mechanistic conclusions regarding why CCM may be beneficial in ATTR-CM cannot be drawn. The ongoing AMY-CCM registry has been designed to address this by collecting CMR data where available, allowing correlation between baseline fibrosis burden and clinical outcomes.

Whether the observed benefits persist over longer follow-up, including sustained improvement in EF, reverse remodeling, and survival, remains to be determined.

Another limitation lies in potential selection bias; participating centers had significant expertise in both heart failure and device therapy, which may not be representative of general practice. Nevertheless, this also ensures procedural quality and strengthens internal validity.

## Figures and Tables

**Table 1:** Socio-demographic characteristics at baseline.

General Characteristics	Total
Total Number (*n*)	10
Age, years, mean [DS]	76.7 ± 4.8
Male gender, number (%)	9 (90)
BMI (kg/m^2^)	24.4 ± 3.4
Years since first diagnosis	3.1 ± 1.7
HF Hospitalization (WHF) in the last 12 months	2.0 ± 1.2
Hypertension (%)	90%
Diabetes (%)	50%
Dyslipidemia (%)	40%
Chronic pulmonary disease (%)	10%
Chronic kidney disease (%)	70%
Chronic liver disease (%)	20%
Coronary artery disease (%)	20%
Atrial fibrillation (%)	50%
Aortic Stenosis (%)	20%

**Table 2:** Pharmacological treatment at baseline.

General Characteristics	Total
ACE-I (%)	30
ARBs (%)	0
BBs (%)	60
Diuretics (%)	100
CCBs (%)	10
Antiplatelets (%)	50
Anticoagulants (%)	50
Anti-arrhythmics (%)	30
Vaso-dilators (%)	0
Digoxin (%)	0
MRAs (%)	40
ARNI (%)	50
SGLUT-2 (%)	20
Tafamidis (%)	30

**Table 3:** Echocardiographic characteristics at baseline.

General Characteristics	Total
NYHA class	3.2 ± 0.4
6MWT (meters)	264.6 ± 100.1
KCCQs	42.0 ± 31
NT pro-BNP (pg/mL)	4300.8 ± 3085.8
eGFR (mL/min/1.73 m^2^)	52.0 ± 8.2

**Table 4:** Clinical characteristics and blood values at baseline.

General Characteristics	Total
EF (%)	36.5 ± 7.3
iLAV (mL/m^2^)	39.7 ± 7.5
LV IVS (mm)	21.3 ± 3.5
LV PP (mm)	18 ± 6.7
E/E′	11.4 ± 7.7
RV free wall thickness (mm)	12.0 ± 5.4
TAPSE (mm)	12.2 ± 2.3
Pericardial effusion (%)	40
Circumferential mid global strain (%)	7.5 ± 1.5

**Table 5:** Patient-by-patient study’s endpoints analysis.

PRE-CCM									POST-CCM				
Pts	Sex	Age	ATTR-CM Wild Type (WT) Hereditary (H)	Time to Diagnosis (Months)	NYHA	EF (%)	Tafamidis	WHF Previous 12 Months	WHF After CCM	NYHA	EF (%)	Tafamidis	Follow-Up (Months)
1	M	70	WT	68	4	36	No	4	0	2	45	Yes	42
2	M	82	WT	16	3	42	No	4	0	2	42	Yes	12
3	M	72	H	79	3	24	No	4	0	2	28	Yes	12
4	M	76	WT	88	3	29	Yes	2	1	2	42	Yes	12
5	M	83	WT	16	3	41	No	2	0	2	38	Yes	12
6	M	78	WT	22	3	30	Yes	2	0	2	39	Yes	12
7	M	57	WT	18	3	33	No	2	0	2	41	Yes	12
8	M	85	WT	34	3	40	No	1	0	2	48	Yes	34
9	M	77	WT	58	3	45	No	2	0	2	45	Yes	39
10	M	81	WT	28	3	45	Yes	1	0	2	45	Yes	19

**Table 6:** Comparison of overall changes before and after CCM implant.

Parameter	Delta
EF (%)	+4.8 ± 6.1
E/E′ (%)	−11.1 ± 22.7
TAPSE (%)	+5.3 ± 10.3
NYHA class	−1.2 ± 0.4
6MWT (%)	+31.3 ± 53.3
KCCQs (%)	+121.0 ± 252.8

In this table are shown the mean changes before and after CCM, expressed in percentage.

## Data Availability

Data will be available on request to the corresponding author.

## Abbreviations

The following abbreviations are used in this manuscript:

ACE-I	Angiotensin-Converting Enzyme Inhibitor
AL	Light-Chain Amyloidosis
AMY-CCM	Amyloidosis–Cardiac Contractility Modulation Registry
ARB	Angiotensin Receptor Blocker
ARNI	Angiotensin Receptor–Neprilysin Inhibitor
ATTR	Transthyretin
ATTR-CM	Transthyretin Cardiac Amyloidosis
BB	Beta-Blocker
BMI	Body Mass Index
CABG	Coronary Artery Bypass Grafting
CaMKII	Calcium/Calmodulin-Dependent Protein Kinase II
CCB	Calcium Channel Blocker
CCM	Cardiac Contractility Modulation
CMR	Cardiac Magnetic Resonance
CRT	Cardiac Resynchronization Therapy
ECG	Electrocardiogram
EDV	End-Diastolic Volume
EF	Ejection Fraction
eGFR	Estimated Glomerular Filtration Rate
ESV	End-Systolic Volume
H	Hereditary
HF	Heart Failure
HSP70	Heat Shock Protein 70
ICD	Implantable Cardioverter-Defibrillator
IQR	Interquartile Range
KCCQ	Kansas City Cardiomyopathy Questionnaire
MAPK	Mitogen-Activated Protein Kinase
MRA	Mineralocorticoid Receptor Antagonist
NT-proBNP	N-terminal pro-B-type Natriuretic Peptide
NYHA	New York Heart Association (Functional Class)
OMT	Optimal Medical Therapy
PCI	Percutaneous Coronary Intervention
PKA	Protein Kinase A
PKB	Protein Kinase B
PLB	Phospholamban
ROS	Reactive Oxygen Species
RyR2	Ryanodine Receptor 2
SERCA	Sarcoplasmic/Endoplasmic Reticulum Calcium ATPase
SGLT2-i	Sodium-Glucose Cotransporter-2 Inhibitor
TAPSE	Tricuspid Annular Plane Systolic Excursion
TTR	Transthyretin Protein
WHF	Worsening Heart Failure
WT	Wild-Type
α-MHC	Alpha-Myosin Heavy Chain
